# Supplementary material for: A Comprehensive Assessment of Ultraviolet-Radiation-Induced Mutations in Flammulina filiformis Using Whole-Genome Resequencing
Source: J Fungi (Basel). 2024 Mar 20;10(3):228. doi: 10.3390/jof10030228 (PMC10971301; doi:10.3390/jof10030228)
Supplement: Supplementary file 1 [file jof-10-00228-s001.zip › Supplementary Material S8/KEGG annotation/out/64381550635650.os/KO/out_map/map05160.html]

KEGG PATHWAY: Hepatitis C - Reference pathway


|  |  |
| --- | --- |
| **Hepatitis C - Reference pathway** |  |

[
Pathway menu
| Organism menu
| Pathway entry
| Show description
| User data mapping
]

Hepatitis C virus (HCV) is a major cause of chronic liver disease. The HCV employ several strategies to perturb host cell immunity. After invasion, HCV RNA genome functions directly as an mRNA in the cytoplasm of the host cell and forms membrane-associated replication complexes along with non-structural proteins. Viral RNA can trigger the RIG-I pathway and interferon production during this process. Translated HCV protein products regulate immune response to inhibit the action of interferon. HCV core and NS5A proteins appear to be the most important molecules with regulatory functions that modulate transcription, cellular proliferation, and apoptosis.

|  |  |
| --- | --- |
| Reference pathway | 100% |
